# Identification of a small pericardial effusion on contrast-enhanced computed tomography indicating cardiac perforation and pericardial injury following blunt trauma: A case report

**DOI:** 10.1016/j.tcr.2021.100515

**Published:** 2021-08-03

**Authors:** Ryo Esumi, Tadashi Kaneko, Yuichi Akama, Toru Shinkai, Yohei Ieki, Saki Bessho, Yu Shomura, Hiroshi Imai

**Affiliations:** a Emergency and Critical Care Center, Mie University Hospital, Japan; b Department of Thoracic and Cardiovascular Surgery, Mie University Hospital, Japan

**Keywords:** Echography, Troponin, Pericardial effusion, Cardiac perforation, Blunt trauma, Computed tomography

## Abstract

Blunt trauma may cause cardiac perforation requiring emergency surgical repair. Cardiac perforations are usually diagnosed by the presence of a pericardial effusion on echocardiography. However, cardiac perforations and pericardial effusions are sometimes too small to detect, resulting in underdiagnosis. In this case report, we describe a 22-year-old man who was involved in a traffic accident, admitted in a state of shock, and was initially treated for tension pneumothorax and liver and spleen injuries. His initial computed tomography scans revealed a small region of enhancement, corresponding to a small pericardial effusion, indicative of a cardiac perforation. Thus, an emergency median sternotomy was performed. He was diagnosed with perforation of the left atrial ear and right atrium, which were repaired surgically. His liver and spleen injuries were also treated, and the patient was discharged 44 days after admission. The detection of a small pericardial effusion on enhanced computed tomography enabled rapid diagnosis of a cardiac perforation and ensured emergency surgical repair could be performed as soon as possible.

**Learning objectives:**

• To acknowledge the difficulty of diagnosing cardiac perforation in patients with pericardial injury, based on conventional signs of blunt cardiac injury, such as sternal fracture, serum cardiac enzymes, and hemothorax.
• To recognize that a small pericardial effusion on enhanced computed tomography scans is an important finding that should raise suspicion of cardiac perforation and pericardial injury.

## Introduction

Cardiac perforation caused by blunt cardiac injury is potentially fatal [1], without surgical repair. It is traditionally diagnosed based on the symptoms of complicated pericardiac effusion. Since around 2000, pericardiac effusion has mostly been diagnosed by cardiac echography and computed tomography. Here, we report a case of cardiac perforation caused by blunt trauma that required surgical repair. Echocardiography was negative, and a small pericardial effusion was detected on computed tomography. Enhancement of the pericardial effusion was a crucial diagnostic clue that prompted surgical treatment of the cardiac perforation. This case highlights the possibility of cardiac perforation caused by blunt trauma, and that the presence of a small pericardial effusion should be considered an important diagnostic clue.

## Case report

**Fig. 1:**
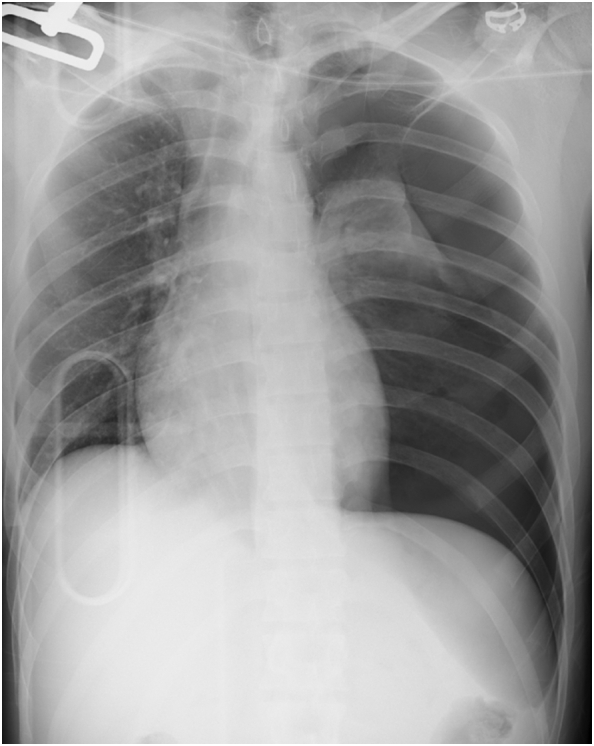
Initial chest X-ray showing left tension pneumothorax. No clear evidence of hemothorax is visible.

**Fig. 2:**
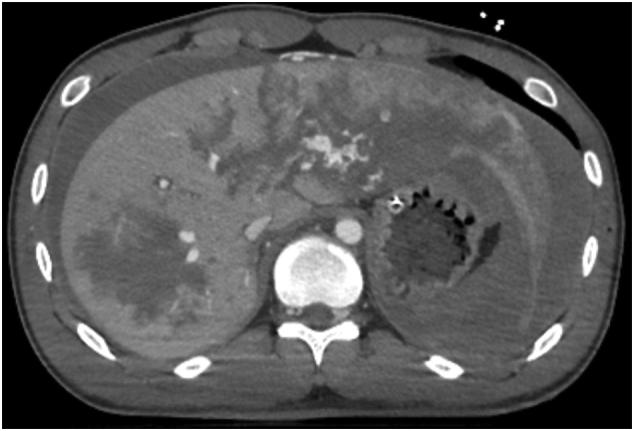
Enhanced abdominal computed tomography showing abdominal bleeding and left and right liver lobe injuries.

**Fig. 3:**
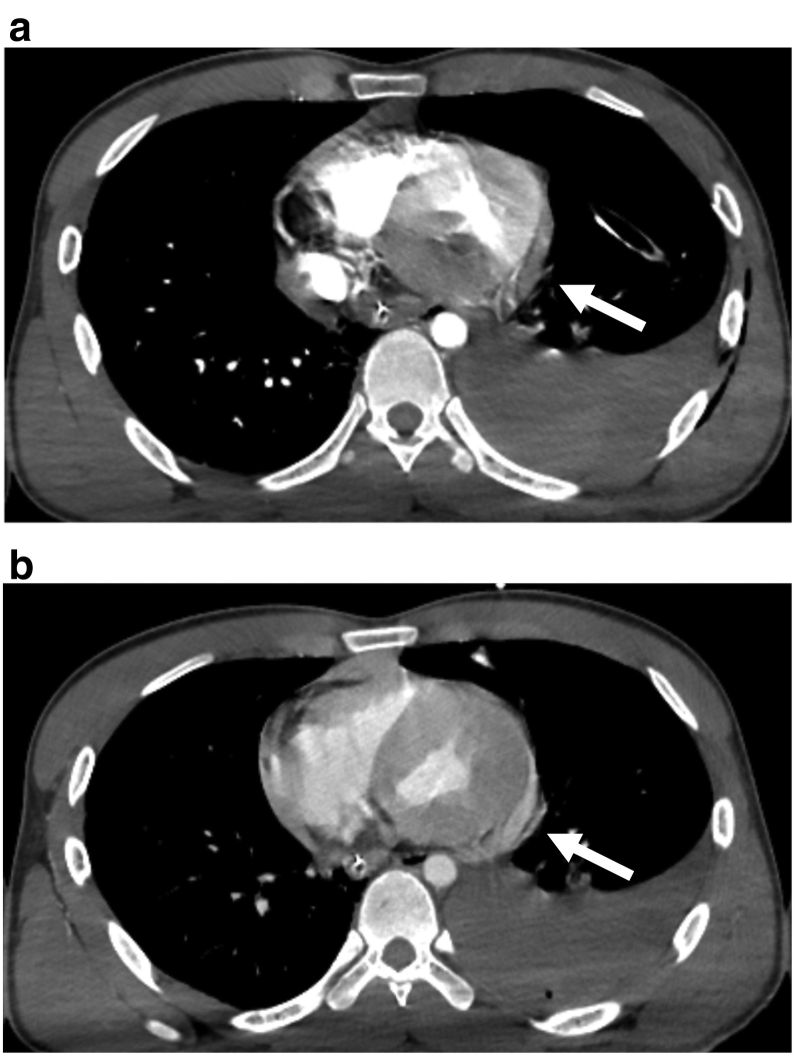
(a) Early-phase enhanced chest computed tomography showing a small pericardial effusion and left hemothorax. (b) Late-phase enhanced chest computed tomography showing an enhanced pericardial effusion compared with the early phase (arrows).

**Fig. 4:**
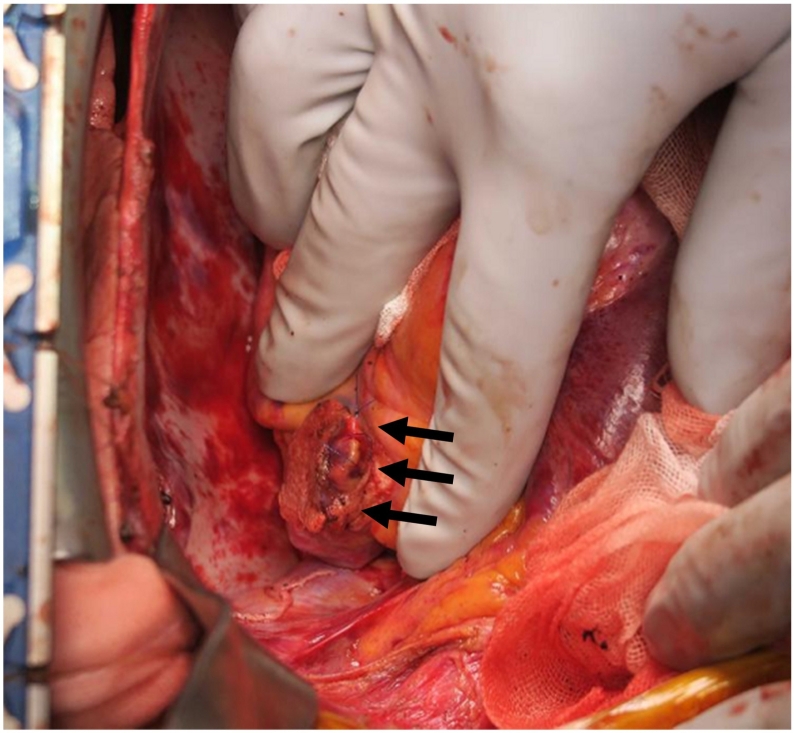
Operative findings of cardiac surgery. The left atrial ear was repaired with proline sutures (arrows).

A 22-year-old man was involved in a traffic accident, consisting of a single head-on collision of his vehicle. He was transported by the emergency medical services to our hospital in a state of traumatic shock, and underwent fluid transfusion. His physical examinations were respiratory rate, 19 breaths/min; heart rate, 158 beats/min; blood pressure, 106/77 mmHg; and a maximum Glasgow coma scale score 15. A portable chest X-ray revealed left tension pneumothorax without hemothorax (Fig. 1). Echography revealed abdominal fluid but was negative for pericardial effusion. After tracheal intubation, insertion of a left chest tube, and initiation of blood transfusion, his systolic blood pressure was maintained above 100 mmHg, and computed tomography and angiography were performed in the angio-computed tomography room. Computed tomography showed liver and splenic injury with abdominal bleeding (Fig. 2), and transarterial embolization was started. After transarterial embolization of the liver and spleen, his systolic blood pressure dropped to 70 mmHg and more than 500 mL of blood was collected via the left chest tube. Slight enhancement, corresponding to a small pericardial effusion, was spotted on the initial computed tomography scans that disappeared after transarterial embolization (Fig. 3), indicative of cardiac perforation with pericardial injury (perforation). Therefore, we performed cardiac repair via median sternotomy together with further gauze packing for the liver and splenic injuries. Left atrial ear, right atrium, and pericardial perforation were diagnosed and repaired (Fig. 4). His serum troponin I level was 7275.1 pg/mL on admission; but this value was only recorded after surgical repair was initiated. Gauze packing and open abdominal management were finished 3 days after admission. Unfortunately, he developed a wound infection as a complication of the median sternotomy, and vacuum-assisted closure was continued between days 21 and 41. He also underwent surgical repair of a fracture of the right ankle on day 30. The patient was discharged on day 44.

## Discussion

Although the present case experienced cardiac perforation following blunt cardiac injury, its diagnosis was delayed because there were no symptoms of cardiac tamponade or pericardial effusion. Cardiac perforation associated with greater pericardial effusion is often easy to diagnose with echocardiography. Other ways to diagnose or predict cardiac perforation following blunt cardiac injury have been reported. Complications of thoracic trauma are a risk factor, and sternal fracture is sometimes associated with blunt cardiac injury. However, sternal fracture is not specific for blunt cardiac injury [2], [3], and rib fracture does not always cause blunt cardiac injury [3], [4]. Serum cardiac enzymes (e.g., troponin, creatine kinase-MB fraction) are associated with mortality and the severity of blunt cardiac injury and blunt trauma [5], [6]. Hemothorax may also predict blunt cardiac injury, although there is insufficient data on what proportion of patients needed surgical repair [7], [8]. In the present case, tension pneumothorax was diagnosed as fatal chest trauma with fractures to a few ribs (left 6–7) without pericardial effusion by echocardiography. Although his serum troponin I level was elevated, this not reported before surgical repair was commenced. These findings highlight the difficulty of diagnosing cardiac perforation with pericardial perforation, especially in cases with nonspecific findings, such as rib fracture, hemothorax, and a very small pericardial effusion that is not visible on echocardiography.

The presence of a small enhanced pericardial effusion on computed tomography is useful for the diagnosis of cardiac perforation caused by blunt cardiac injury. It has been reported that, although enhanced computed tomography imaging is not possible in all cases of blunt cardiac injury, cardiac perforation caused by blunt cardiac injury could be diagnosed in cases with leakage from the cardiac wall or enhanced pericardial effusion [9], [10]. Similarly, cardiac perforation with pericardial injury that does not show sufficient pericardial effusion for detection by echocardiography could be diagnosed based on enhancement of a small pericardial effusion. For surgical repair of cardiac perforation, early recognition and limited fluid resuscitation are extremely important. In the present case, spotting an enhanced pericardial effusion was crucial to quickly recognize a difficult-to-diagnose cardiac perforation and commence surgical repair.

## Conclusion

We diagnosed cardiac perforation caused by pericardial injury in a patient with blunt trauma by spotting an enhanced pericardial effusion on computed tomography; this finding supported our decision to perform surgical repair.

## Declaration of competing interest

None.

## References

[1] Hanschen M., Kanz K.G., Kirchhoff C. (2015). Blunt cardiac injury in the severely injured – a retrospective multicentre study. PLoS One.

[2] Heidelberg L., Uhlich R., Bosarge P., Kerby J., Hu P. (2019). The depth of sternal fracture displacement is not associated with blunt cardiac injury. J. Surg. Res..

[3] Joseph B., Joker T.O., Khalil M. (2016). Identifying the broken heart: predictors of mortality and morbidity in suspected blunt cardiac injury. Am. J. Surg..

[4] Hammer M.M., Raptis D.A., Cummings K.W. (2016). Imaging in blunt cardiac injury: computed tomographic findings in cardiac contusion and associated injuries. Injury.

[5] Kalbitz M., Pressmar J., Stecher J. (2017). The role of troponin in blunt cardiac injury after multiple trauma in human. World J. Surg..

[6] Emet M., Akoz A., Aslan S., Saritas A., Cakir Z., Acemoglu H. (2010). Assessment of cardiac injury in patients with blunt chest trauma. Eur. J. Trauma Emerg. Surg..

[7] Grigorian A., Milliken J., Livingston J.K. (2019). National risk factors for blunt cardiac injury: hemopneumothorax is the strongest predictor. Am. J. Surg..

[8] Tran H.V., Charles M., Garrett R.C., Kempe P.W., Howard A., Khorgami Z. (2020). Ten-year trends in traumatic cardiac injury and outcomes: a trauma registry analysis. Ann. Thorac. Surg..

[9] Koweek L.M.H., Chung J.H., Ghoshhajra B.B. (2020). ACR appropriateness criteria ® blunt chest trauma-suspected cardiac injury. J. Am. Coll. Radiol..

[10] Baxi A.J., Restrepo C., Mumbower A., McCarthy M., Rashmi K. (2015). Cardiac injuries: a review of multidetector computed tomography findings. Trauma Mon..

